# Supplementary material for: Gill-associated ammonia oxidizers are widespread in teleost fish
Source: Microbiol Spectr. 2024 Sep 26;12(11):e00295-24. doi: 10.1128/spectrum.00295-24 (PMC11537070; doi:10.1128/spectrum.00295-24)
Supplement: Supplemental material — Tables S1 and S2. [file spectrum.00295-24-s0001.docx]

**Supplementary figures**

**Table S1:** Gill microbiome studies analyzed for presence of gill-associated nitrifiers. For each study, accession number and the fish species are provided, as well as the environment from which the samples were collected and the sequencing method used. The salinity of the surrounding water of the fish and the sampling method by which the gill samples were collected are also recorded, together with the primers used to target the 16S rRNA gene, as well as references to the original studies.

| **Bioproject accession number** | **Fish species** | **Environment** | **Sequencing method** | **Salinity** | **Gill sampling method** | **Region targeted (primers)** | **Reference** |
| --- | --- | --- | --- | --- | --- | --- | --- |
| PRJNA488363 | Rainbow trout | Laboratory (flowthrough) | MiSeq | FW | Whole | V1-V3 (28F, 519R) | Brown et al., 2019 |
| PRJNA401167 | 53 tropical reef fish species | Wild | MiSeq | SW | Whole | V4 (515F, 806R) | Pratte et al., 2018 |
| PRJEB32307 | Atlantic salmon | Aquaculture (cage) | MiSeq | FW SW | Mucus + adherent to tissue | V4 (515F, 806R) | Birlanga et al., 2022 |
| PRJNA565540 | Atlantic salmon | Laboratory (RAS) | MiSeq | SW | Whole | V1-V3 (28F, 519R) | Brown et al., 2021 |
| PRJDB10074 | Rainbow trout | Laboratory (RAS) | MiSeq | FW | Whole | V3-V4 (341F, 806R) | Takeuchi et al., 2021 |
| PRJNA396452 | Yellowtail kingfish | Aquaculture (cage) | MiSeq | SW | Swabs | V1-V2 (27F, 338R) | Legrand et al., 2018 |
| PRJNA531247 | Mediterranean fish | Wild | MiSeq | SW | Whole | V4-V5 (515F, 926R) | Ruis-Rodriguez et al., 2020 |
| PRJNA531247 | Myctophid (lantern)fish | Wild | HiSeq | SW | Whole | V3-V4 (341F, 805R) | Gallet et al., 2019 |
| PRJEB27458 | Pacific chub mackerel | Wild | MiSeq | SW | Swabs | V4 (515F, 806R) | Minich et al., 2020 |
| PRJNA575053 | Seabass | Aquaculture (open water) | MiSeq | SW | Swabs | V4 (515F, 806R) | Rosado et al., 2019 |
| PRJEB37803 | Rabbitfish | Wild | HiSeq | SW | Whole | V3-V4 (338F, 806R) | Wu et al., 2020 |
| PRJEB36802 | Atlantic salmon | Aquaculture (RAS & FT) | MiSeq | FW | Swabs | V4 (515F, 806R) | Minich et al., 2020 |
| PRJEB36802 | Southern Bluefin tuna | Aquaculture (cage) | MiSeq | SW | Swabs | V4 (515F, 806R) | Minich et al., 2020 |
| PRJEB36802 | Yellowtail kingfish | Aquaculture (RAS & FT & cage) | MiSeq | SW | Swabs | V4 (515F, 806R) | Minich et al., 2021 |
| PRJNA601603 | Silver carp + bighead carp | Aquaculture (lake) | HiSeq | FW | Whole | V3-V4 (341F, 806R) | Kuang et al., 2020 |
| PRJNA687505 | Seabass + seabream | Aquaculture (open water) | MiSeq | SW | Swabs | V4 (515F, 806R) | Rosado et al., 2021 |
| PRJNA667072 | Atlantic salmon | Aquaculture (cage) | MiSeq | SW | Swabs + tissue biopsy | V3-V4 (341F, 806R) | Clinton et al., 2021 |
| PRJNA248305 | Rainbow trout | Aquaculture (flowthrough) | Roche 454 | FW | Whole | V1-V3 (A17F, 519R) | Lowrey et al., 2015 |
| PRJEB54736 | 101 fish species | Wild | MiSeq + NovaSeq | SW | Whole | V4 (515F, 806R) | Minich et al., 2022 |
| PRJNA518052 | Grass carp + southern catfish | Laboratory (RAS) | HiSeq | FW | Whole | V4-V5 (515F, 907R) | Zhang et al., 2019 |
| PRJNA632679 | Indian river fish | Wild | HiSeq | FW | Whole | V3-V4 (341F, 805R) | Malakar et al., 2021 |
| PRJNA692072 | Seabream | Aquaculture (cage) | MiSeq | SW | Whole | V3-V4 (341F, 785R) | Quero et al., 2022 |
| PRJNA557254 | Rainbow trout | Laboratory | MiSeq | FW | Whole | V3-V4 (341F, 785R) | Valdes et al., 2020 |
| PRJNA839167 + PRJNA839174 | Amazonian flag cichlid (Mesonauta festivus) | Wild | MiSeq | FW | Whole | V3-V4 (347F, 803R) | Sylvain et al., 2022 |
| PRJNA847066 | Rainbow trout, Nile tilapia, grey mullet, Atlantic salmon | Laboratory (flowthrough) Aquaculture (pond), Laboratory (RAS) | NovaSeq | FW | Swabs + tissue + wash in RT | V4 (515F, 806R) | Clokie et al., 2022 |
| PRJNA784534 | Rainbow trout | Laboratory (flowthrough) | MiSeq | FW | Whole | V3-V4 (341F, 785R) | Bellec et al., 2022 |
| PRJNA826829 | Atlantic salmon | Aquaculture (FW RAS & SW cage) | MiSeq | FW + SW | Swabs | V3-V4 (341F, 785R) | Lorgen-Ritchie et al., 2022b |
| PRJNA663352 | Atlantic salmon | Laboratory (flowthrough) | MiSeq | SW | Swabs | V3-V4 (341F, 785R) | Bledsoe et al., 2022 |
| PRJNA649054 | Atlantic salmon | Laboratory (flowthrough) | MiSeq | SW | Swabs + tissue | V1-V3 (27F, 519R) | Slinger et al., 2021 |
| PRJNA855906 | Goldfish | Laboratory (flowthrough) | MiSeq | FW | Whole | V4 (515F, 806R) | Ortiz et al., 2022 |
| PRJNA601439 | Rainbow trout | Laboratory (RAS) | MiSeq | FW | Whole + sorted bacterial biomass | V1-V3 (28F, 519R) | Xu et al., 2020 |
| PRJNA748412 | Sparidae species | Wild | MiSeq | SW | Mucus scraped | V3-V4 (341F, 805R) | Scheifler et al., 2022 |
| PRJNA796496 | Cobia | Aquaculture (tank) | MiSeq | SW | Whole | V4 (515F, 806R) | Marcela Villegas-Plazas et al., 2022 |
| PRJNA302804 | Atlantic salmon | Laboratory (RAS) | HiSeq | FW | Whole | V6 (967F 1064R) | Schmidt et al., 2016 |
| PRJNA748818 | Grass carp | Laboratory (pond) | PacBio | FW | Swabs | V1-V6 (27F, 1492R) | Zhao et al., 2022 |
| PRJNA702883 | Marine medaka | Laboratory (aquarium) | MiSeq | SW | Whole | V3-V4 (341F, 806R) | Lai et al., 2022 |
| PRJNA770664 | Nile tilapia + grey mullet | Aquaculture (pond) | NovaSeq | FW | Swabs | V3-V4 (341F, 806R) | Elsheshtawy et al., 2021 |
| PRJNA514914 | Anglerfish | Wild | MiSeq | SW | Whole | V4 (515F, 806R) | Freed et al., 2019 |
| PRJNA304614 | Red snapper | Wild | 454 Roche | SW | Whole | V1-V3 (27F, 519R) | Tarnecki et al., 2016 |
| PRJNA816828 | Largemouth bass | Aquaculture (RAS) | MiSeq | FW | Whole | V3-V4 (338F, 806R) | Zhang et al., 2022 |
| PRJEB40530 | Spotted seabass | Aquaculture (pond) | MiSeq | SW | Swabs | V4-V5 (515F, 909R) | Ye et al., 2021 |
| PRJNA763808 | Discus fish | Laboratory (tank) | MiSeq | FW | Mucus | V3-V4 (341F, 806R) | Huang et al., 2022 |
| PRJNA768447 | Two endemic species in Sao Fernando river | Wild & aquaculture (flowthrough) | MiSeq | FW | Whole | V3-V4 (341F, 785R) | Damasceno et al., 2022 |
| PRJNA881007 | Atlantic salmon | Aquaculture (RAS + flowthrough & RAS only) | MiSeq | FW | Swabs | V1-V3 (27F, 519R) | Quezada-Rodriguez et al., 2023 |

**Table S2:** Gill microbiome studies with gill-associated nitrifiers present. For each study, the fish species is provided, together with the salinity of the surrounding water of the fish and the sampling method by which the gill microbiome was assessed. The number of nitrifier ASVs present in the gills of each species is provided (gill-associated), as well as the number of nitrifier ASVs that were only found in the gill and not the environment. For each study, the associated study accession numbers are also provided.

| **Fish species** | **Salinity** | **Nr. of gill-associated nitrifier ASVs** | **Nr. of gill-specific nitrifier ASVs** | **Sampling method** | **Reference** |
| --- | --- | --- | --- | --- | --- |
| Rainbow trout (Oncorhynchus mykiss) | FW | 1 *Nitrosospira* | N/A | Whole | Lowrey et al., 2015 |
| Atlantic salmon (Salmo salar) | FW | 12 *Nitrosomonas* | 0 | Whole | Schmidt et al., 2016 |
| Yellowtail kingfish (Seriola lalandi) | SW | 1 *Nitrosomonas* | 1 *Nitrosomonas* | Swabs | Legrand et al., 2018 |
| 53 tropical reef fish species | SW | 2 *Nitrosomonas* | 0 | Whole | Pratte et al., 2018 |
| Rainbow trout | FW | 1 *Nitrosomonas* | 1 *Nitrosomonas* | Whole | Brown et al., 2019 |
| Grass carp (Ctenopharyngodon idella) and southern catfish (Silurus meridionalis) | FW | 6 *Nitrosomonas* in southern catfish, 4 in grass carp | 1 *Nitrosomonas* (in southern catfish) | Whole | Zhang et al., 2019 |
| Atlantic salmon | FW | 11 *Nitrosomonas*, 1 *Nitrosospira* | 2 *Nitrosomonas* | Swabs | Minich et al., 2020 |
| Rabbitfish | SW | 3 *Nitrosomonas* | All gill-specific | Whole | Wu et al., 2020 |
| Rainbow trout | FW | 1 *Nitrosomonas* | N/A | Whole and sorted bacterial biomass | Xu et al., 2020 |
| Atlantic salmon | SW | 1 *Nitrosomonas* | 0 | Swabs + tissue biopsy | Clinton et al., 2021 |
| Nile tilapia (Oreochromis niloticus) and grey mullet (Mugil cephalus) | FW | 29 *Nitrosomonas* in Nile tilapia, 2 *Nitrosospira* in Nile tilapia, 4 *Nitrosospira* in Grey mullet | All gill-specific | Swabs | Elsheshtawy et al., 2021 |
| Yellowtail kingfish | SW | 6 *Nitrosomonas*, 2 *Nitrosospira* | 1 *Nitrosomonas*, 1 *Nitrosospira* | Swabs | Minich et al., 2021 |
| Sea bass (Dicentrarchus labrax) and sea bream (Sparus aurata) | SW | 2 *Nitrosomonas* in Seabass, 1 in Seabream (1 ASV shared), 1 *Nitrosospira* in seabream | 0 | Swabs | Rosado et al., 2021 |
| Atlantic salmon | SW | 1 *Nitrosomonas* | N/A | Swabs + tissue biopsy | Slinger et al., 2021 |
| Spotted seabass  (Lateolabrax maculatus) | SW | 4 *Nitrosomonas* | 2 *Nitrosomonas* | Swabs | Ye et al., 2021 |
| Rainbow trout | FW | 14 *Nitrosomonas* | 3 *Nitrosomonas* | Biopsy | Bellec et al., 2022 |
| Atlantic salmon | FW  SW | 1 *Nitrosomonas*, 1 *Nitrosospira* | N/A | Swabs + adherent to tissue | Birlanga et al., 2022 |
| Atlantic salmon | SW | 14 *Nitrosomonas*, 5 *Nitrosospira* | 5 *Nitrosomonas*, 1 *Nitrosospira* | Swabs | Bledsoe et al., 2022 |
| Rainbow trout, Nile tilapia, grey mullet, Atlantic salmon | FW | 27 *Nitrosomonas* in Nile tilapia, 7 *Nitrosomonas* in Rainbow trout, 4 *Nitrosomonas* in Atlantic salmon, 1 *Nitrosomonas* in Grey mullet. 2 *Nitrosospira* in Nile tilapia, 3 *Nitrosospira* in Grey mullet | All gill-specific | Swabs + tissue biopsy + wash | Clokie et al., 2022 |
| Marine medaka (Oryzias melastigma) | SW | 1 *Nitrosomonas* | N/A | Whole | Lai et al., 2022 |
| Atlantic salmon | FW  SW | 8 *Nitrosomonas*, 2 *Nitrosospira* | 1 *Nitrosospira* | Swabs | Lorgen-Ritchie et al., 2022 |
| 101 fish species | SW | 1 *Nitrosospira* | 0 | Whole | Minich et al., 2022 |
| Sparidae species | SW | 4 *Nitrosomonas* | All gill-specific | Mucus scraped | Scheifler et al., 2022 |
| Amazonian flag cichlid (Mesonauta festivus) | FW | 3 *Nitrosomonas* | All gill-specific | Whole | Sylvain et al., 2022 |
| Largemouth bass (Micropterus salmoides) | FW | 1 *Nitrosomonas*, 1 *Nitrosospira* | 1 *Nitrosomonas* | Whole | Zhang et al., 2022 |
| Atlantic salmon | FW | 17 *Nitrosomonas*, 3 *Nitrosospira* | 4 *Nitrosomonas*, 2 *Nitrosospira* | Swabs | Quezada-Rodriguez et al., 2023 |
| Total |  | **215** | **115** |  |  |
| Total Nitrosospira |  | 29 | 16 |  |  |
| Total Nitrosomonas |  | 186 | 99 |  |  |
